# A Dynamin-Actin Interaction Is Required for Vesicle Scission during Endocytosis in Yeast

**DOI:** 10.1016/j.cub.2015.01.061

**Published:** 2015-03-30

**Authors:** Sarah E. Palmer, Iwona I. Smaczynska-de Rooij, Christopher J. Marklew, Ellen G. Allwood, Ritu Mishra, Simeon Johnson, Martin W. Goldberg, Kathryn R. Ayscough

**Affiliations:** 1 Department of Biomedical Science, University of Sheffield, Sheffield S10 2TN, UK; 2 Department of Biological Science, Durham University, Durham DH1 3LE, UK

## Abstract

Actin is critical for endocytosis in yeast cells, and also in mammalian cells under tension. However, questions remain as to how force generated through actin polymerization is transmitted to the plasma membrane to drive invagination and scission. Here, we reveal that the yeast dynamin Vps1 binds and bundles filamentous actin. Mutational analysis of Vps1 in a helix of the stalk domain identifies a mutant RR457-458EE that binds actin more weakly. In vivo analysis of Vps1 function demonstrates that the mutation disrupts endocytosis but not other functions of Vps1 such as vacuolar trafficking or peroxisome fission. The mutant Vps1 is stably expressed in cells and co-localizes with the endocytic reporters Abp1 and the amphiphysin Rvs167. Detailed analysis of individual endocytic patch behavior indicates that the mutation causes aberrant movements in later stages of endocytosis, consistent with a scission defect. Ultrastructural analysis of yeast cells using electron microscopy reveals a significant increase in invagination depth, further supporting a role for the Vps1-actin interaction during scission. In vitro analysis of the mutant protein demonstrates that—like wild-type Vps1—it is able to form oligomeric rings, but, critically, it has lost its ability to bundle actin filaments into higher-order structures. A model is proposed in which actin filaments bind Vps1 during invagination, and this interaction is important to transduce the force of actin polymerization to the membrane to drive successful scission.

## Highlights

• Yeast dynamin Vps1 forms oligomeric rings that bind and bundle F-actin in vitro
• Mutations in the Vps1 stalk region disrupt actin binding and bundling
• The Vps1 RR-EE actin binding mutant has defects in endocytic scission in vivo

## Graphical Abstract

**Figure:**
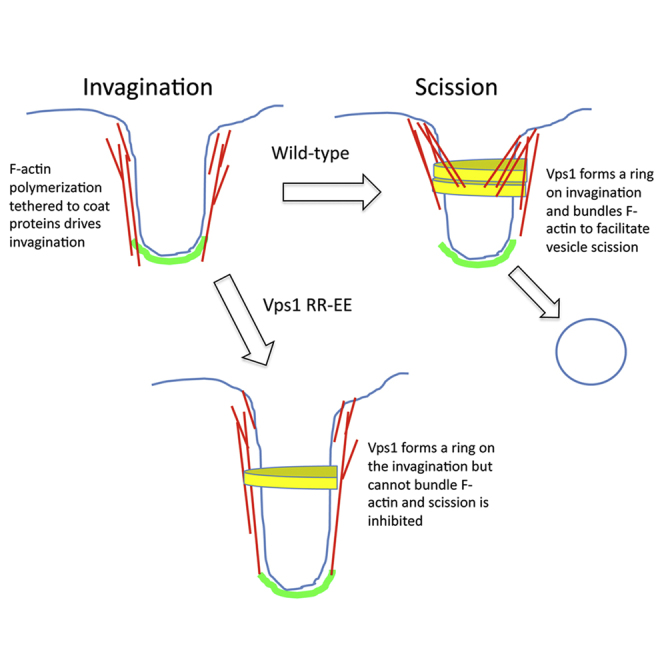

## Teaser

Palmer et al. reveal that the yeast dynamin Vps1 is able to form oligomeric rings that can directly interact with and bundle F-actin. Defects in the Vps1-actin interaction confer endocytic scission defects in vivo.

## Introduction

Live-cell analysis in budding yeast has led the way in understanding the role of actin during endocytosis. Actin is recruited and polymerized through the function of the nucleating Arp2/3 complex and its activator Las17/WASP at specific sites on the plasma membrane, most likely determined by concentrations of endocytic coat proteins and cargo [1–4]. Growth of actin filaments by polymerization, and their movement by type 1 myosins (Myo3 and Myo5), then drives the formation of a pronounced invagination, around which further factors assemble to bring about constriction, scission, and release of the vesicle [2, 4].

Imaging of fluorescently tagged reporters indicates that a similar sequential recruitment and disassembly of protein complexes occurs during mammalian endocytosis. While actin appears to serve an essential function when the plasma membrane is under tension [5], several studies indicate that actin is recruited to the majority of endocytic sites [6, 7]. Recent evidence suggests a positive feedback loop functions at endocytic sites, with dynamin, actin, and N-BAR proteins co-operating to effect efficient scission of membranes to release a vesicle [8]. Quantitative fluorescence microscopy of dynamin-2 revealed that, while low levels of dynamin were present early in endocytosis, actin recruitment directly preceded the precise temporal recruitment of further dynamin, sufficient to generate a single dynamin ring at sites, prior to scission [9]. While the interdependency of dynamin and actin is made clear by these studies, the mechanisms underlying these observations remains unknown. Another study has demonstrated a direct interaction between dynamin and actin, but while this was shown to be required for stress fiber formation in podocytes, mutations affecting dynamin actin interaction did not inhibit transferrin uptake suggesting that the direct interaction was not required for endocytosis [10].

While the role of actin in driving membrane invagination in yeast is clear, its role in scission has not been closely studied. Scission function has been attributed to the amphiphysin dimer Rvs161/Rvs167 and to the dynamin homolog Vps1, with both proteins localizing to endocytic sites immediately prior to scission [11]. The Rvs167 SH3 domain binds directly to Vps1, and in vivo a reduced level of amphiphysin is recruited to endocytic sites in the absence of *vps1* [12]. When both components are deleted, >65% of invaginations show retraction back toward the plane of the plasma membrane indicative of a scission defect [11].

Vps1 is one of three dynamin-like proteins in yeast but is the only one of these dynamins that functions in membrane trafficking pathways. Like other dynamins, Vps1 is predicted to have a GTPase domain and a stalk region. However, in common with non-classical dynamins it does not have a PH domain but an alternative domain referred to as Insert B. Despite the absence of the PH domain, Vps1 can bind and tubulate liposomes [11]. The localization, interactions, and deletion phenotypes of Vps1 suggest that it is likely to function in a similar way to Dynamin-1 or Dynamin-2 in mammalian endocytosis [11, 13–15]. In addition to a function in endocytosis, Vps1 also functions at other stages of membrane trafficking including Golgi to vacuole (lysosome) transport and endosomal recycling [16–23]. Recently, Dynamin-2 has also been reported to function in a range of membrane trafficking events beyond endocytosis raising the possibility that dynamin function is more promiscuous than previously considered, and that dynamins may act as general scission factors on multiple membranes, possibly regulated by distinct components at each site [16–23].

In this study, we have used both in vitro and in vivo approaches to address key questions about the functional interactions between actin and dynamin to gain mechanistic insight into the first membrane fission step of the endocytic pathway.

## Results

**Figure 1:**
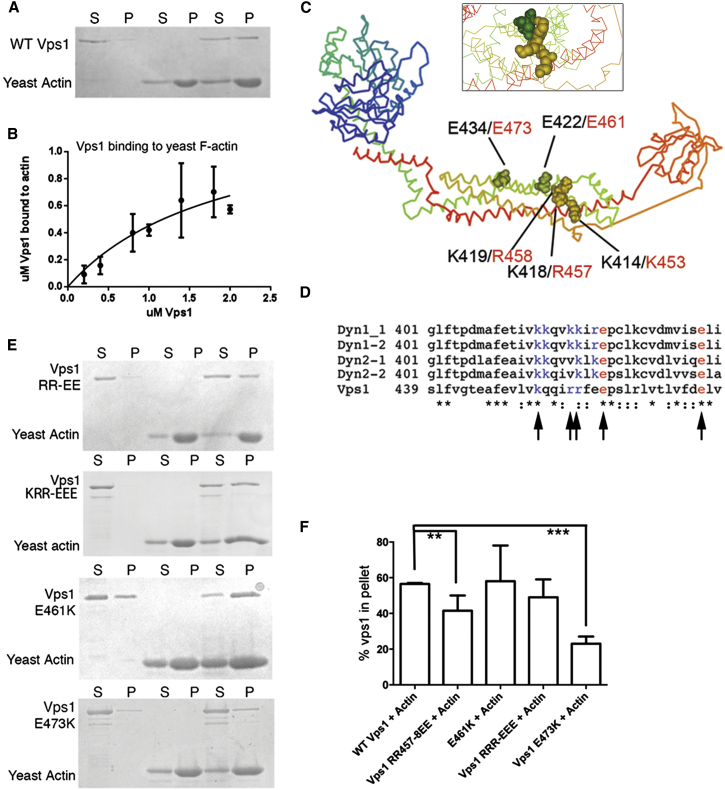
Wild-Type and Mutant Vps1 Interact with Actin (A) Vps1 (1.5 μM) was incubated with 3 μM yeast F-actin before high-speed centrifugation. Pellets and supernatants of samples containing Vps1 alone, actin alone, or Vps1+actin were separated by SDS-PAGE. (B) Densitometry analysis of multiple actin pelleting assays with a range of Vps1 concentrations allowed generation of a binding curve to calculate binding affinity. Error bars are SEM. (C) Crystal structure of Dynamin (PDB 3SNH) with dynamin positions denoted in black for equivalent mutagenized Vps1 residues (red). Inset reveals mutagenized residues projecting away from the stalk domain. (D) Sequence alignment of stalk region shown to bind actin in Dynamin-1. Accession numbers in sequence order: NP004399.2, NP001005336.1, NP001005360, NP001005361, Vps1 CAA82071. Vps1 residues mutated in this study are highlighted and shown with arrows. Blue represents basic residues and red represents acidic residues mutated. (E) Vps1 mutants incubated with yeast F-actin before centrifugation. Pellets and supernatants were separated by SDS-PAGE. (F) Vps1 in the pellet was analyzed using densitometry. n ≥ 3 for each mutant. Error bar is SE. Asterisks indicate level of statistical significance of differences compared to wild-type control; p value from unpaired t test 0.009 for RR-EE and 0.0001 for E473K.

A direct dynamin-actin interaction has previously only been reported for mammalian dynamin-1; thus, an initial step was to determine whether the interaction is conserved with yeast dynamin Vps1 and actin. Recombinant Vps1 was purified, incubated with F-actin, followed by centrifugation. Binding was assessed with both yeast actin (Figure 1A) and rabbit muscle actin (Figure S1A). An increase in amount of Vps1 associating with F-actin in the pellet was observed (Figure 1B; Figures S1B and S1E). The average K_D_ of Vps1 from four independent experiments for yeast actin was calculated to be 1.9 ± 0.36 μM (K_D_ for rabbit actin 0.92 ± 0.31 μM).

Having demonstrated that Vps1 binds to actin, we aimed to determine whether mutations that compromise the Dynamin-1-actin interaction also affect the Vps1-actin interaction. Based on the crystal structure of dynamin, the suggested actin binding region lies along a helix that forms part of the central stalk domain [24, 25]. Based on primary and secondary structure comparisons, this helix in Vps1 is well conserved making selection of charge-switch mutations straightforward (Figures 1C and 1D). All selected residues are predicted to project outward from the stalk region and were considered unlikely to disrupt secondary structure of the region. Mutants RR457,458EE (RR-EE), K453E R457E R458E (KRR-EEE), E461K, and E473K were generated both in plasmids and integrated into the yeast genome.

Wild-type and mutant Vps1 were expressed, purified, and incubated with yeast F-actin and centrifuged to determine extent of binding (Figure 1E). Quantification of Vps1 pelleting (n ≥ 3 independent experiments) indicated that there was a significant reduction in actin binding of the RR-EE and the E473K mutants (p value from unpaired t test 0.009 for RR-EE; 0.0001 for E473K; Figure 1F). A similar pattern of binding occurred following incubation with rabbit muscle actin (data not shown). The mutations were predicted to not affect folding but to determine whether other functions of Vps1 were affected; lipid binding was also assessed. As we have shown previously, wild-type Vps1 is able to bind to liposomes [11]. This binding interaction did not appear to be compromised in any of the Vps1 mutants (Figure S1C).

**Figure 2:**
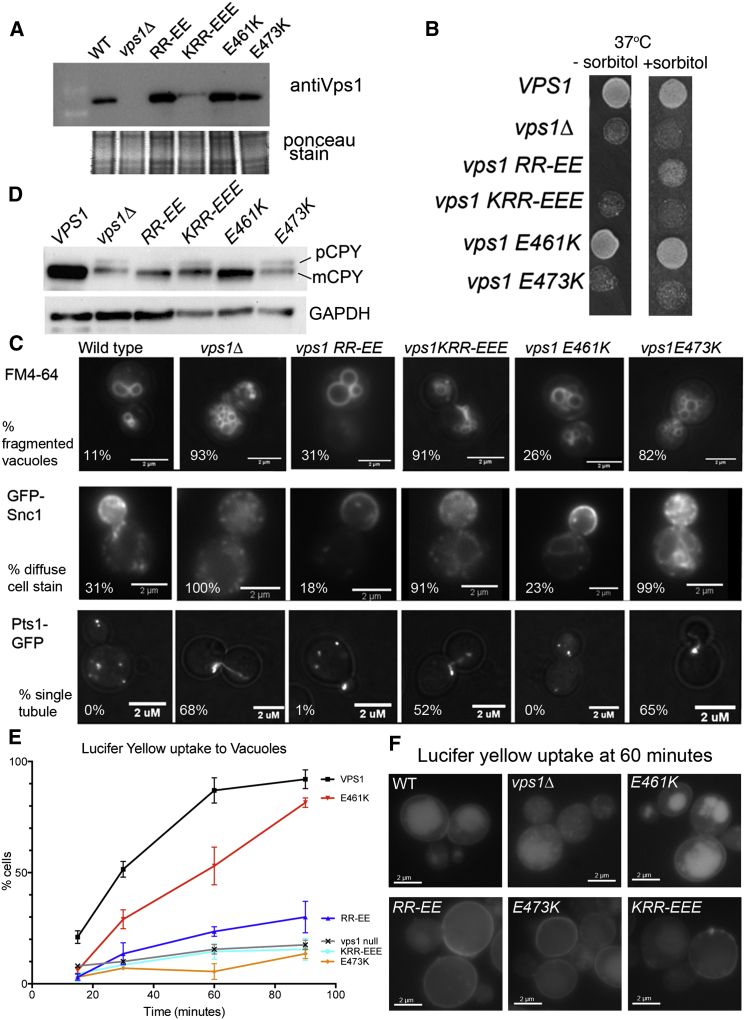
The Effect of Vps1 Mutations on Functions Requiring Vps1 (A) Whole-cell extracts from yeast expressing mutated Vps1, separated by SDS-PAGE, blotted, and probed with anti-Vps1 antibodies. Apart from KRR-EEE, all mutants expressed at normal levels. Loading control is Ponceau-stained blot. (B) The effect of mutations on growth at a range of temperatures for cells expressing integrated *vps1* mutants. Shown is growth at 37°C in the presence and absence of the osmotic support molecule sorbitol. (C) Vps1 is required for normal vacuolar morphology, recycling of the snare Snc1 through endosomes, and for peroxisomal fission. The effect of mutations on these functions was assessed using FM4-64, GFP-Snc1, and GFP-PTS1, respectively, and representative data are shown in upper, middle, and lower panels. (D) Carboxypeptidase Y normally functions in the vacuole where it is cleaved to its mature form (mCPY). In the absence of *vps1*, and in the KRR and K473E mutants, some of this material accumulates in a precursor form (pCPY). Below is a GAPDH loading control. (E) Uptake of the fluid phase marker lucifer yellow was analyzed after incubation at 21°C for up to 90 min. Localization of the majority of the dye to vacuoles (large round or lobed structure) was counted. n = 100 cells in each of two independent experiments. Errors are SD. (F) Images of cells at the 60 min time point with lucifer yellow.

Analysis of phenotypes arising from the mutations was then performed in vivo. The four mutants, a deletion, and a wild-type strain were tested for protein expression and overall growth phenotypes. All four mutants expressed in yeast with only the KRR-EEE mutant showing reduced levels compared to wild-type (Figure 2A). Deletion of *vps1* causes a temperature-sensitive phenotype with cells unable to grow at 37°C [11, 23]. The mutants gave distinct phenotypes in growth assays with the KRR-EEE, RR-EE, and E473K mutations causing temperature sensitivity, while E461K-expressing cells rescued temperature sensitivity of the deletion strain. Sorbitol is able to rescue some temperature-sensitive phenotypes, especially those associated with actin defects in endocytosis [26]. When sorbitol was present on plates at 37°C, the RR-EE mutant now showed growth, while the two other temperature-sensitive mutants showed no clear improvement in growth (Figure 2B).

To determine whether the mutants affected some or all phenotypes associated with *vps1* deletion, assays were performed including analysis of vacuole morphology (*vps1*Δ cells have a class F phenotype; with a single large vacuole surrounded by multiple small fragmented vacuoles, while wild-type cells usually contain two to five similarly sized vacuoles [22]); endosomal recycling of SNARE reporter Snc1-GFP (mutants in endosomal recycling fail to show clear plasma membrane staining of the reporter [27]); and peroxisome fission in cells carrying a reporter GFP-PTS1 (*vps1*Δ have a single elongated peroxisome while wild-type cells have multiple small peroxisomes [20]). In all these assays, two mutants, RR-EE and E461K, behaved similar to the wild-type *VPS1* strain, while two mutants, KRR-EEE and E473K, behaved like the strain lacking *vps1* (Figure 2C). Carboxypeptidase Y, an enzyme that is cleaved to its mature form (mCPY) in the vacuole, is less efficiently processed in the *vps1* deletion and a precursor accumulates (pCPY) [22, 23]. As shown (Figure 2D), pCPY accumulates in the KRR-EEE and E473K mutants, indicating that they share this trafficking defect with the *vps1* deletion strain.

Finally, analysis of lucifer yellow (LY) uptake was performed to assess fluid phase endocytosis (Figures 2E and 2F). Wild-type cells internalize LY by endocytosis and traffic it to the vacuole, while *vps1* deletion cells accumulate the dye in vesicles and endosomes. Interestingly, RR-EE and E461K mutants that behaved similarly to wild-type cells in other assays, showed defects in their ability to traffic LY to the vacuole. Analysis of the proportion of cells with LY predominantly at the plasma membrane, endosomes, or vacuole at 60 min is shown in Figure S2. Together, these data indicate that KRR-EEE and E473K mutants behave largely as non-functional proteins with all in vivo phenotypes indistinguishable from cells completely lacking Vps1. The Vps1 E461K mutant does not significantly affect actin binding and behaves like wild-type Vps1 in most cell assays, with just a delay in endocytosis. In contrast, the mutation RR457-458EE revealed a specific endocytosis defect in vivo and reduced actin binding in vitro. A more detailed analysis of endocytic defects in the vps1 RR-EE mutant would allow us to understand the functional links between actin binding and endocytosis more clearly. However, in order to corroborate the data from the F-actin binding assays (Figures 1E and 1F), it was important to first substantiate the in vitro result with affinity measurements. Therefore, further binding assays were undertaken with the vps1 RR-EE mutant to measure the actin binding affinity. This analysis revealed an affinity of vps1 RR-EE for yeast actin, with a K_D_ of 4.8 ± 2.6 μM (compared to 1.9 μM for wild-type), supporting the previous data that this mutant has a reduced binding capacity for actin (Figure S1D).

**Figure 3:**
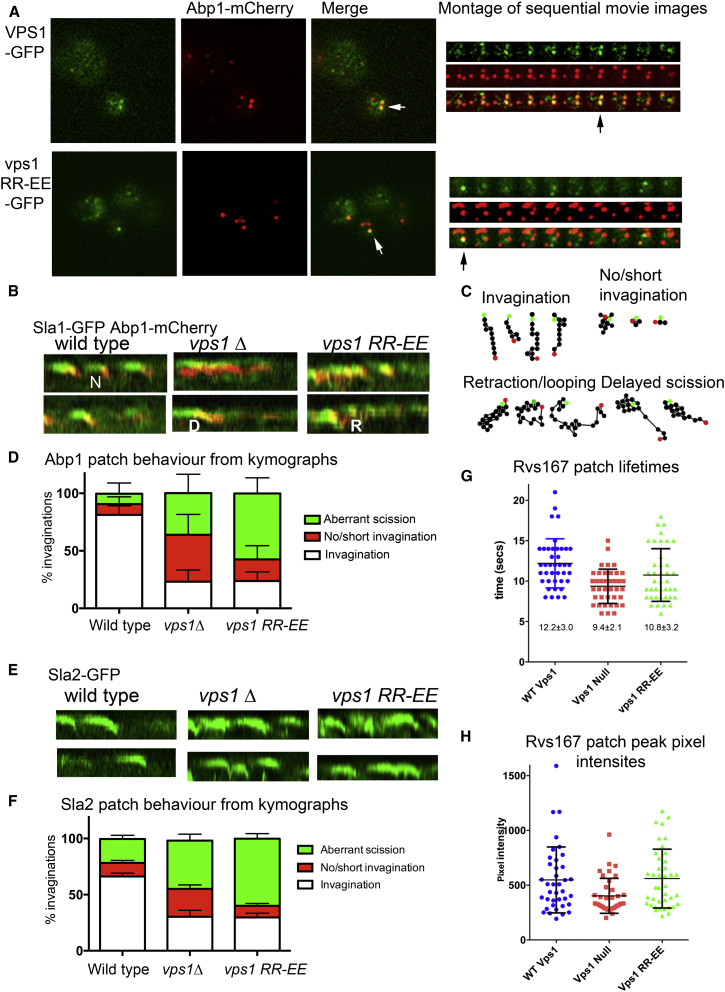
The Effect of Vps1 RR-EE Mutation on Localization and Individual Endocytic Events (A) Wild-type and RR-EE Vps1 tagged with GFP on plasmids under control of the Vps1 promoter were transformed into cells also expressing the endocytic reporter Abp1 tagged with mCherry. Cells were visualized in TIRF. White arrows indicate colocalized patches. A montage from a movie for each is shown to the right. Black arrow indicates the time from which the left image was recorded. (B) Sla1-GFP (endocytic coat marker) Abp1-mCherry (actin marker) were analyzed in live cells. Shown are representative 60-s kymographs. N shows a normal invagination, R indicates example of retraction, and D delayed scission. (C) The behavior of patches was analyzed from kymographs and by generation of patch tracks. Shown are representative patch tracks for categories of Abp1 patch behaviors. Green spots indicate start of track and red spots the end. (D) Abp1 patches were categorized as showing normal invagination, no or short invagination, or normal invagination followed by aberrant scission. This latter category included retraction and delayed scission. Shown is analysis of nine or more patches in ≥17 cells from two independent experiments. Error SD. The endocytic coat protein Sla2 was also analyzed for its behavior at endocytic sites. (E) Representative 90 s kymographs from time-lapse movies of Sla2 in cells expressing wild-type or Vps1 RR-EE. (F) Graph summarizing patch behavior. Shown is analysis of ≥22 patches in seven or more cells from two independent experiments. Error is SD. (G) Lifetimes of Rvs167-GFP at endocytic sites were recorded (≥40 patches in ten cells for each strain). Shown is average lifetime ±SE. In a one-way ANOVA, lifetime is significantly different between wild-type and null (p < 0.05) but not the RR-EE mutant. (H) Peak pixel intensity of each spot was recorded as a measure of the maximum level of Rvs167 recruited to the site (±SE). One way ANOVA indicates Rvs167 intensities in the null is significantly reduced compared to cells with wild-type or vps1 RR-EE mutant (p ≥ 0.05).

Given that functions other than endocytosis were rescued in cells expressing the vps1 RR-EE mutant, it was first important to establish whether this mutant protein is able to localize to sites of endocytosis or whether endocytosis was defective due to lack of Vps1 recruitment to these sites. Two approaches were used to demonstrate cortical patch localization. First, colocalization of Vps1-GFP (WT or mutant protein) with the reporter protein Abp1 was analyzed using TIRF microscopy. As shown in Figure 3A, clear puncta could be seen for both WT and mutant Vps1 proteins, and these showed co-localization in a temporal manner with Abp1 at the cell cortex (% co-localization wild-type 24% ± 4.1% SEM n = 8 cells; vps1 RR-EE 21.5% ± 5.1% SEM, n = 7). This relatively low co-localization is similar to that observed previously and probably reflects difficulties with incorporation of GFP tagged Vps1 into functionally relevant structures (see later analysis). Second a bimolecular fluorescence complementation (BiFC) approach was used in which each half of the yellow fluorescent protein variant Venus was appended to either Vps1 or the RR-EE mutant (V_N_) or to the amphiphysin Rvs167, which functions at endocytic sites but not on endosomes (V_C_). As shown (Figure S3), Rvs167-V_C_ and Vps1-V_N_ show clear punctate sites of interaction at the cell cortex in an organization indicative of endocytic sites. Again, the Vps1 RR-EE mutant exhibited similar localization to the wild-type protein.

Reporter proteins that assemble at the endocytic site have been widely used to determine whether mutations or deletions cause defects at distinct stages of endocytosis [28, 29]. Previously, we demonstrated that deletion of *vps1* caused a defect in both invagination and scission events [11, 12]. Analysis of proteins Sla1-GFP, Abp1-mCherry, Sla2-GFP, and Rvs167-GFP was used to gain further insights to the stage of endocytosis that is defective in the vps1 RR-EE mutant. Kymograph analysis of co-expressed Sla1-GFP and Abp1-mCherry indicated that the endocytic coat assembled appropriately in cells but that there were defects in invagination and scission in the *vps1*-null and mutant strain. Due to the highly aberrant behaviors observed, timing of patch lifetimes was considered unreliable, and instead the behavior of individual patches was categorized as normal invagination; no/short invagination; or aberrant scission. This latter class included patches that appeared to invaginate but then either retract back toward the plasma membrane (retraction) and those that remained invaginated for several seconds before reporters disassembled (delayed scission). Examples of these behaviors as patch tracks are shown on kymographs and patch tracks (Figures 3B and 3C). A summary graph of the Abp1-mCherry patch behaviors is shown (Figure 3D). Both the *vps1*-null and the *vps1 RR-EE*-expressing strain show fewer normal invaginations compared to those in wild-type cells (in multiple t test p ≤ 0.0001). Interestingly, the *vps1 RR-EE* mutant showed a higher proportion of aberrant scission than the *vps1*-null strain (p ≤ 0.0002 in t test), while the null strain showed increases in both invagination and scission defects. The behavior of an endocytic coat protein Sla2 was also analyzed in cells expressing wild-type and mutant Vps1 (Figure 3E for kymographs). Again, the mutant appeared to show a distinct phenotype, with patches in the RR-EE mutant mostly showing invagination from the plane of the membrane followed by a retraction or other aberrant movement before disassembly. A summary graph of Sla2-GFP patch behavior is shown (Figure 3F). As with Abp1, the proportion of scission defects is greater in the mutant compared to the null strain (p value ≤ 0.0001 in t test).

Previously, we demonstrated that deletion of *vps1* led to a reduction in the Rvs167 signal at the endocytic site suggesting that Vps1 might play a role in stabilizing Rvs167 at sites or might facilitate its oligomerization [12]. The lifetime and intensity of the Rvs167 signal in the *vps1* mutant was analyzed and compared to *VPS1* wild-type and null strains. As shown in Figures 3G and 3H, the vps1 RR-EE mutant shows no significant differences from wild-type in intensity or lifetime of Rvs167-GFP staining, indicating that amphiphysin can be recruited and maintained correctly in the mutant strain.

**Figure 4:**
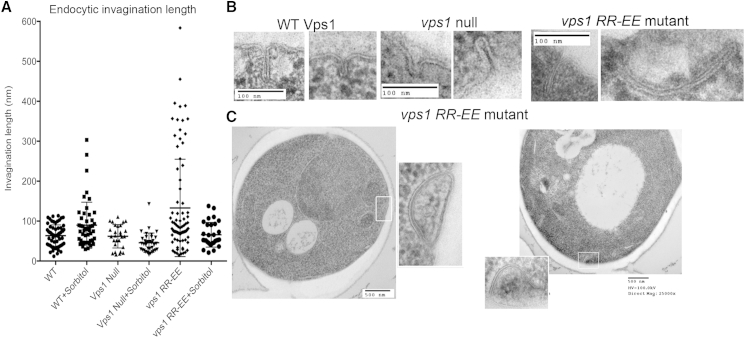
The Effect of the Vps1 RR-EE Mutation on Ultrastructure of Endocytic Invaginations (A) Cells expressing wild-type or mutant Vps1 proteins were grown to log phase and processed for electron microscopy. At least 30 invaginations in at least 100 cell sections were analyzed for each strain. The effect of sorbitol on invagination length was analyzed with invagination length measured for ≥25 invaginations for each strain. All data points are shown and error bars are SD. Reduction in invagination length in the presence of sorbitol is significant in an unpaired t test (p = 0.036). (B) Examples of invaginations in wild-type and mutant cells. (C) Examples of long invaginations in the vps1 RR-EE mutant that appear to show curvature of the tubule back to the membrane.

While observation of retracting endocytic patches is indicative of a scission problem, clearer insight into the defect caused by *vps1 RR-EE* mutation used electron microscopy to analyze invaginations. As shown (Figure 4), this approach revealed a dramatic increase in the length of invaginations observed in cells expressing the RR-EE mutant, with an average invagination length of 133 nm compared to 61 nm in wild-type cells (n = 84, vps1 RR-EE; n = 64 WT p value ≤ 0.0001 unpaired t test). Given that invagination lengths were only measured when full membrane profiles were visible, lengths in excess of 200 nm are likely to be under-represented in the data. Interestingly, many invaginations in the RR-EE mutant bend and in some cases curve back toward the plasma membrane, with examples observed of possible re-fusion at the membrane (Figure 4C).

Studies from other labs have demonstrated the formation of long invaginations at the plasma membrane in cells in which eisosomal formation has been compromised [30]. To determine whether the long invaginations observed here are associated with defects in eisosome formation, Pil1-mRFP was visualized in wild-type and vps1 RR-EE-expressing cells. As shown (Figure S4), eisosomal size and organization appear to be the same in both strains, indicating the long invaginations are not due to eisosomal defects. Given that a bimodal distribution of invaginations was observed in the RR-EE-expressing cells, it is possible that the long invaginations represent activation of alternative endocytic pathways [31, 32]. However, even if these longer invaginations (≥200 nm) are excluded from calculations, there is still a significant increase in invagination length in the RR-EE mutants (75 nm compared to 61 nm in wild-type p ≥ 0.01).

Previously, we demonstrated that addition of osmoremedial growth medium containing sorbitol reduced the actin requirement during yeast endocytosis, suggesting actin polymerization was required to generate sufficient inward force to invaginate the membrane against the outward force of turgor pressure [26]. Subsequently, mammalian cells with membranes under tension were also shown to require actin for endocytosis [5]. If the interaction between dynamin and actin is required to transduce force for scission, as well as invagination, then addition of sorbitol may facilitate scission when such an actin-based force has been disrupted. Electron microscopy (EM) analysis was therefore undertaken with cells incubated with sorbitol. Most strikingly, the high proportion of long invaginations in vps1 RR-EE-expressing cells is completely rescued under this condition (Figure 4A) with the mean invagination length now 65 nm (n = 30).

**Figure 5:**
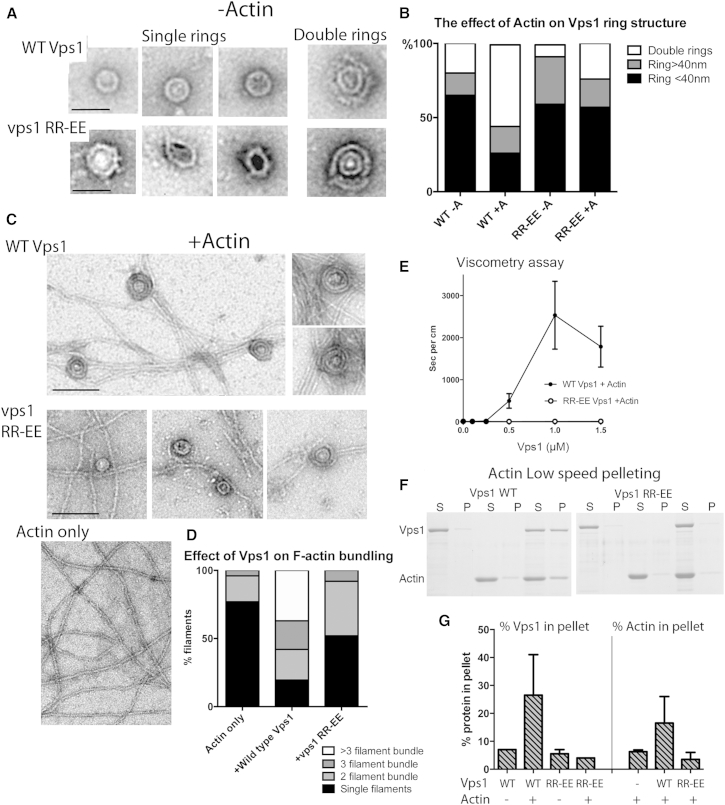
Mutant Vps1 Is Impaired in Binding and Bundling of F-Actin (A) Vps1 was purified and visualized by EM. Shown are examples of single- and double-ring structures for wild-type and vps1 RR-EE. (B) The proportion of single and double-ring structures in the presence and absence of actin. Rings with diameter >40 nm with less regular structure were called loose rings. (C) EM images of actin alone or following incubation with wild-type or mutant Vps1. (D) In the presence of Vps1, actin was reorganized with an increased incidence of bundles. Single filaments or bundles with two, three, or more than three actin filaments were counted. (E) A falling-ball viscometry assay was performed with increasing concentrations of wild-type or mutant Vps1. Shown is a plot of the rate of fall of the ball for each concentration of Vps1. Error bars are SE; n = 3 independent experiments. (F) A low-speed actin pelleting assay with wild-type and mutant Vps1. (G) The proportion of both Vps1 (left) and actin (right) in the pellets from three independent pelleting assays was assessed using densitometry. Error is SD.

The data presented indicate that an actin-dynamin/Vps1 interaction is conserved and that mutation of residues disrupting this interaction cause a specific defect in endocytic scission. To increase our understanding of how the interaction contributes to scission, we investigated the interaction between Vps1 and actin further. Vps1 was purified and analyzed using EM. For the first time, we are able to reveal that, like mammalian dynamin-1, Vps1 is able to form an oligomeric ring structure (Figure 5A). The Vps1 rings have a diameter of about 32 nm (±3.7 nm SD). This size is slightly smaller than that reported for the dynamin-1 ring, which is proposed to contain 13 dimers in a ring of about 43 nm. Intriguingly, a smaller proportion (∼20%) of the Vps1 structures visualized have a structure in which a second ring appears to wrap around the first. We have called this a double-ring structure. Within a double ring, the diameter of the inner ring is maintained, while the outer ring diameter is about 63 nm (±8 nm). In addition to these defined single- and double-ring structures, about 15% of the rings were slightly larger (>40 nm) and less well defined or regular. These we termed loose rings. Purified Vps1 RR-EE was able to form single rings, though these were often less regular than wild-type rings. Fewer double rings were observed, and these second rings were sometimes fragmented (Figure 5A). The average diameter of the single-ring structures was similar to wild-type (35 ± 4.7 nm). To determine whether the mutation was affecting the structure of the protein, circular dichroism analysis was undertaken (Figure S5). This analysis indicated that the secondary structure of the protein is fully maintained in the vps1 RR-EE mutant.

The effect of incubating Vps1 with actin was then analyzed. Two distinct effects were observed when incubating wild-type Vps1 with actin. First the actin filaments became bundled and crosslinked. Second, the number of oligomeric arrangements of Vps1 (double rings) increased to 55% of structures counted (Figures 5B and 5C). Thus, both actin and Vps1 appear to impact on structural organization of the other, supporting the interplay observed in live-cell studies [8, 9]. The extent of bundling was determined by counting actin in individual fields as single filaments, two, or three or more aligned filaments. In the presence of Vps1, the proportion of actin in bundles of two or more filaments increased from 23 to >80% with 58% in bundles of three or more filaments (Figure 5D). In the case of Vps1 RR-EE, an increase in the proportion of double-ring structures was observed, though this was only to a level of 24% of total structures observed. Most striking, however, was the lack of effect of the mutant protein on actin organization with only 8% of actin filaments in bundles of three or more (Figure 5D).

Because EM only allows protein that forms defined structures to be visualized, the occurrence of actin bundling effects was corroborated using other methods. Both a viscometry assay and a low-speed centrifugation approach were used to determine the effect of wild-type or mutant Vps1 on the extent of actin crosslinking. As shown in Figure 5E, Vps1 addition greatly retarded the passage of a ball through a capillary containing polymerized actin. Reduction in viscosity at the highest Vps1 concentration is possibly indicative of greater bundling that generates channels in the filament mix thus allowing less impeded movement of the ball [33]. The mutant protein did not affect the rate of fall in a capillary compared with actin alone indicating that it was unable to crosslink the filaments.

While single actin filaments are pelleted at speeds of ∼300,000 × *g*, only crosslinked or bundled actin can be pelleted at speeds of 10–15,000 × *g*. As shown in Figures 5F and 5G, both actin and wild-type Vps1 were able to pellet at low speeds, while neither actin nor the vps1 RR-EE mutant showed an increase in the pellet fraction when co-incubated. Taken together, these data indicate that actin filaments can induce or stabilize a higher-order dynamin structure, and that dynamin in turn is able to interact with multiple filaments and generate a crosslinked network. The RR-EE mutation reduces actin binding, and consequently an actin network is unable to form.

## Discussion

In this study, we sought to determine whether the yeast endocytic dynamin Vps1 was able to bind to actin and whether binding was required for all or just a subset of Vps1 functions in membrane trafficking. Strikingly, the ability of Vps1 to bind actin is conserved and appears to involve the same region as in the mammalian dynamin-1 protein [10]. Most importantly, mutational analysis reveals that altering the Vps1-actin interaction causes specific defects in endocytosis but not in other functions of Vps1.

An important finding is that Vps1, like its mammalian counterparts, can form a ring structure. The ring is slightly smaller than that shown for dynamin-1 (32 versus 40 nm) but at the level of EM appears remarkably similar [34, 35]. While incubation with actin appears to induce or stabilize a double-ring structure, the organization of Vps1 within this structure is not currently known and awaits further, structural analysis. The importance of the ring structure for Vps1 function may explain why attempts to localize Vps1 tagged with GFP as the sole form of Vps1 in cells have failed to observe it at endocytic sites, while studies co-expressing tagged and untagged protein have detected such localization [11]. The presence of a large C-terminal tag on all Vps1 molecules might well be expected to impact negatively on ring formation.

Analysis of Vps1 mutants identified a key site (RR457,458) as critical for both actin interaction and endocytic function, while two other mutations (KRR-EEE and E473K) led to defects affecting all functions of Vps1 in addition to endocytosis. The RR-EE mutant protein is unable to bundle actin filaments in vitro and causes a defect in scission in vivo. Together with earlier data, we propose that Vps1 is recruited to the invaginated membrane where it binds directly to actin. This then triggers increased recruitment and stabilization of Vps1 oligomeric structures, potentially equivalent to the increase in dynamin localization observed just before scission in mammalian clathrin-mediated endocytosis [8, 9]. In addition, the Vps1 rings bind and bundle actin filaments at its site of localization. This re-organization of actin may facilitate a switch in the site of force transduction from the tip of the invagination where the force was used to drive the inward membrane movement [36], to the point at which scission is required, on the side of the invagination. Our data provide evidence for the previously postulated idea that force on the membrane itself could be an important contributory factor in the vesicle scission step [21, 37]. If the interaction between Vps1 and actin is reduced, as in the vps1 RR-EE mutant, such that the actin network can no longer be re-organized, force will not be transduced to the appropriate scission site, thus precluding release of the vesicle. This model also suggests that a scission defect would only be predicted to occur in cell types that require the force generated by actin for endocytosis due to membrane tension. Further studies in cell types requiring actin of endocytosis will allow the importance of the direct dynamin-actin interaction to be examined further in mammalian cells. In light of the recent reports of the interplay between these proteins from live-cell analyses, it seems likely that a dynamin-actin interaction will form part of the functional protein network that forms prior to vesicle scission.

Overall, these data provide strong evidence for a mechanism for endocytic vesicle scission in which dynamin brings about a re-organization of the actin network at endocytic sites to allow the force from actin polymerization to drive membrane rearrangement culminating in scission.

## Experimental Procedures

Unless stated otherwise, chemicals were obtained from Sigma-Aldrich, Fisher Scientific, or Formedium. Yeast strains and plasmids used in this study are listed in the Supplemental Information. Point mutations in *VPS1* were generated using site directed mutagenesis (Agilent) with template plasmids pKA677 and pKA850. All strains carrying tags have growth properties similar to control strains. Bimolecular fluorescence complementation (BiFC) assays used strains carrying Venus constructs crossed for co-expression of both N- and C-terminal halves of Venus. Expression levels of Vps1 were measured from western blots of whole-cell extracts using anti-Vps1 antibody [11]. Carboxypeptidase Y processing was analyzed from cell extracts [12]. Pre-cleaned CPY antibodies (Chemicon International) were used at 1:100 dilution and GAPDH antibodies at 1:5,000.

### Biochemical Approaches

Wild-type *VPS1* and *vps1* mutants were expressed in *E. coli* (C43) (Lucigen Overexpress C43(DE3) SOLOs) as His tag fusions and were purified [11]. Vps1 was either imaged directly for EM analysis, actin bundling assays, and circular dichroism analysis or pre-spun at 90,000 × *g*, 15 min at 4°C. Unless stated, purified Vps1 for in vitro assays was from the resulting supernatant, the concentration of which was usually between 1 and 5 μM.

G-actin was purified from rabbit muscle [38] and yeast lysates [39]. Purified rabbit or yeast F-actin was polymerized for 1 hr at room temperature (21°C) with 10% KME (pH 8) (500 mM KCl, 10 mM MgCl_2_, 10 mM EGTA, 100 mM Imidazole [pH 8.0]) and left overnight at 4°C. Purified Vps1 and F-actin were mixed and incubated for 15 min then spun 90,000 × *g*. Supernatant and pellet were separated by SDS-PAGE. Quantification was by densitometry (Bio-Rad ImageLab3.0). K_D_ was calculated using a single site binding curve equation in GraphPad Prism 6. K_D_ from three or more independent experiments was averaged. Falling-ball assays were performed with rabbit actin [38]. To analyze Vps1 and actin using electron microscopy, 1 μM Vps1 was added to 1.5 μM F-actin and incubated for 15 min. 5 μl of a 1:10 dilution of mix was adsorbed on carbon-coated grids, and proteins were visualized by negative staining with uranyl formate. Electron micrographs were recorded using Gatan MultiScan 794 charge-coupled device (CCD) camera on Philips CM100 electron microscope.

#### Cell Biology

Epifluorescence microscopy was performed using Olympus IX-81 microscope with DeltaVision RT Restoration Microscopy with 100× 1.40 numerical aperture oil objective and Photometrics Coolsnap HQ camera. Imaging and image capture was performed using SoftWoRxTM (Applied Precision Instruments). Experiments were carried out at 21°C. For uptake of FM4-64, 0.25 μl of 16 mM FM4-64 was added to 500 μl culture for 90 min. Following washing, z stack images were collected with step sizes of 0.2 μm. For lucifer yellow uptake, cells were incubated with lucifer yellow (Fluka: 13 mg/ml final concentration) for up to 90 min. Cells were washed in buffer (50 mM succinate, 20 mM NaN_3_ [pH 5]) before imaging [40]. For peroxisomal fission, cells were transformed with GFP-PTS1. For live-cell imaging, cells were visualized in synthetic medium. Time-lapse live-cell imaging of GFP-tagged Sla2 and Rvs167 was performed with 1 s time-lapse and 0.5 s exposure. For cells with both Sla1-GFP and Abp1-mCherry time lapse was 1.5 s with 0.25-s exposure for both. For Abp1mCherry alone microscopy was performed using Nikon Eclipse Ti microscope with 100× oil objective and Andor Zyla sCMOS camera. Imaging and image capture was performed using NIS Elements 4.20.01 software. Images were 60 ms exposure with frame interval 0.11 s for total 60 s. TIRF microscopy used Nikon TIRF with 100× oil objective and Photometrics Evolve EMCCD camera. Image capture used NIS Elements 4.20.01 software. Images were 60 s with 2-s interval. All image data sets were deconvolved. Statistical analysis of lifetimes was performed using GraphPad Prism.

Preparation and analysis of yeast for electron microscopy was performed as described previously [41]. Cells were harvested and frozen in a Leica EMPACT high-pressure freezer followed by freeze substitution. Following processing, sections were stained (1% uranyl acetate, then Reynold’s lead citrate). Cells were viewed at 100 kV in a Hitachi H7600 transmission electron microscope. Lengths of invaginations were measured using ImageJ software.

## Author Contributions

S.E.P. designed and performed experiments and data analysis for Figures 1, 2, 3, 5, and S1; I.I.S.-d.R. performed experiments and analysis for Figures 3, S3, and S4; C.J.M. designed, performed, and analyzed data for Figures 5 and S5; E.G.A. made initial findings on which the project was based and was involved in subsequent experimental design; R.M., S.J., and M.W.G. performed experiments and data analysis for Figure 4. K.R.A. undertook experimental design and data analysis for Figures 2, 5, and S2 and wrote the manuscript.

## Supplemental Information

Document S1. Supplemental Experimental Procedures, Figures S1–S5, and Tables S1 and S2

Document S2. Article plus Supplemental Information
