# Supplementary material for: A Dynamin-Actin Interaction Is Required for Vesicle Scission during Endocytosis in Yeast
Source: Curr Biol. 2015 Mar 30;25(7):868–78. doi: 10.1016/j.cub.2015.01.061 (PMC4386032; doi:10.1016/j.cub.2015.01.061)
Supplement: Document S1. Supplemental Experimental Procedures, Figures S1–S5, and Tables S1 and S2 [file mmc1.pdf]

Current Biology

Supplemental Information

A Dynamin-Actin Interaction Is Required for Vesicle Scission during Endocytosis in Yeast

Sarah E. Palmer, Iwona I. Smaczynska-de Rooij, Christopher J. Marklew, Ellen G. Allwood, Ritu Mishra, Simeon Johnson, Martin W. Goldberg, and Kathryn R. Ayscough

Supplemental Information

Supplemental Figures

Figure S1. Biochemical Analysis of Vps1-actin interaction.
Figures are further controls and analysis for data shown in Figure 1

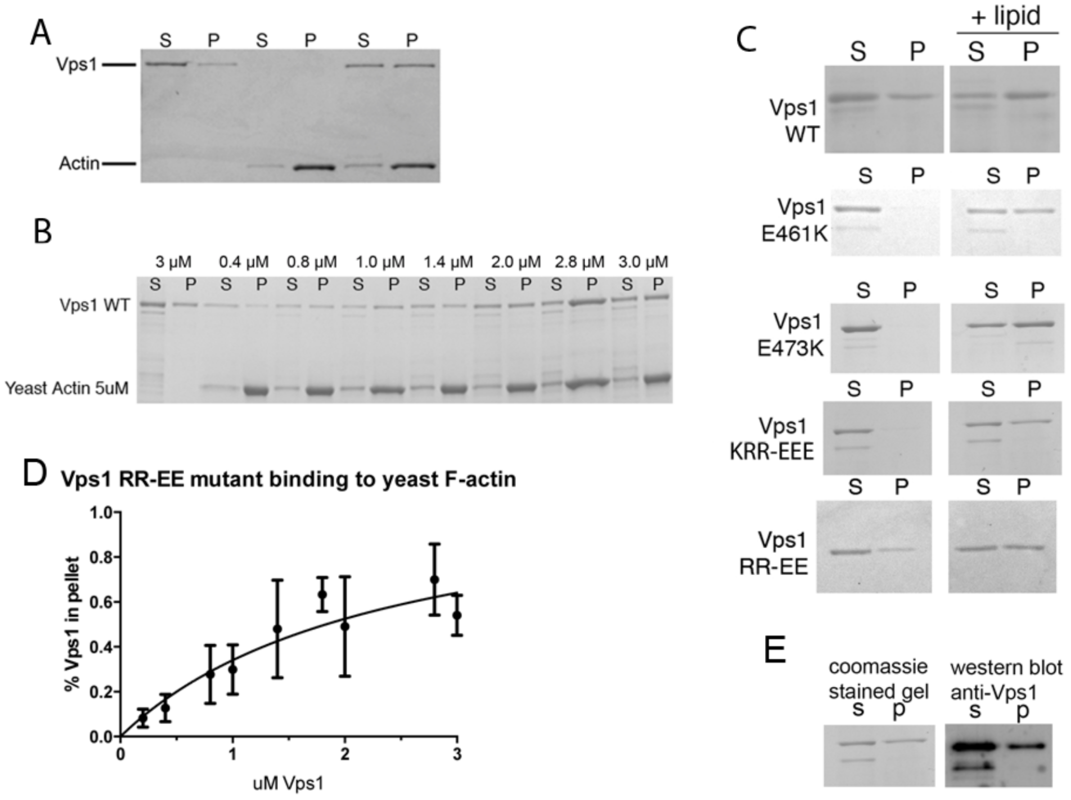

(A) Vps1 was purified as described and 1.5 μM Vps1 was incubated with 3 μM pre-polymerized rabbit muscle F-actin before being subjected to high speed centrifugation. The resulting pellets and supernatants of samples containing Vps1 alone, actin alone or Vps1+ actin were then separated by SDS-PAGE. (B) Vps1 from 0.4 – 3.0 μM was incubated alone or in the presence of 5 μM yeast F-actin. Samples were centrifuged at high speed and samples run as supernatant or pellet fractions. The lower band is a breakdown product of Vps1, see E below. (C) Wild type and mutant Vps1 proteins were incubated with liposomes as described followed by pelleting to determine binding. All mutants were able to bind liposomes indicating binding was not compromised by the mutation. (D) Vps1 was purified as described and 1.5 μM Vps1 RR-EE mutant was incubated with 3 μM pre-polymerized yeast F-actin before being subjected to high speed centrifugation. The resulting pellets and supernatants of samples containing Vps1 alone, actin alone or Vps1+ actin were

then separated by SDS-PAGE. Densitometry analysis of multiple actin pelleting assays (n=4) with a range of wild-type Vps1 concentrations allowed a binding curve to be generated to calculate binding affinity. (E) On occasion there appeared to be a breakdown product of Vps1 visible on Coomassie gels. To demonstrate that this was indeed Vps1 and not a contaminant, western blotting was performed. The samples in this assay were spun to investigate pelleting in the presence of actin. The lower (degradation) band does not pellet. However the contribution of both N- and C-termini to overall dynamin folding would suggest that this form of the protein is unlikely to be structured rather than indicating a particular binding site.

Figure S2. The effect of *vps1* mutants at 60 minutes in a Lucifer Yellow assay.

Data relates to time course data for Figure 2E. Data shown is a further analysis of LY localization at the 60 minute time point from this analysis.

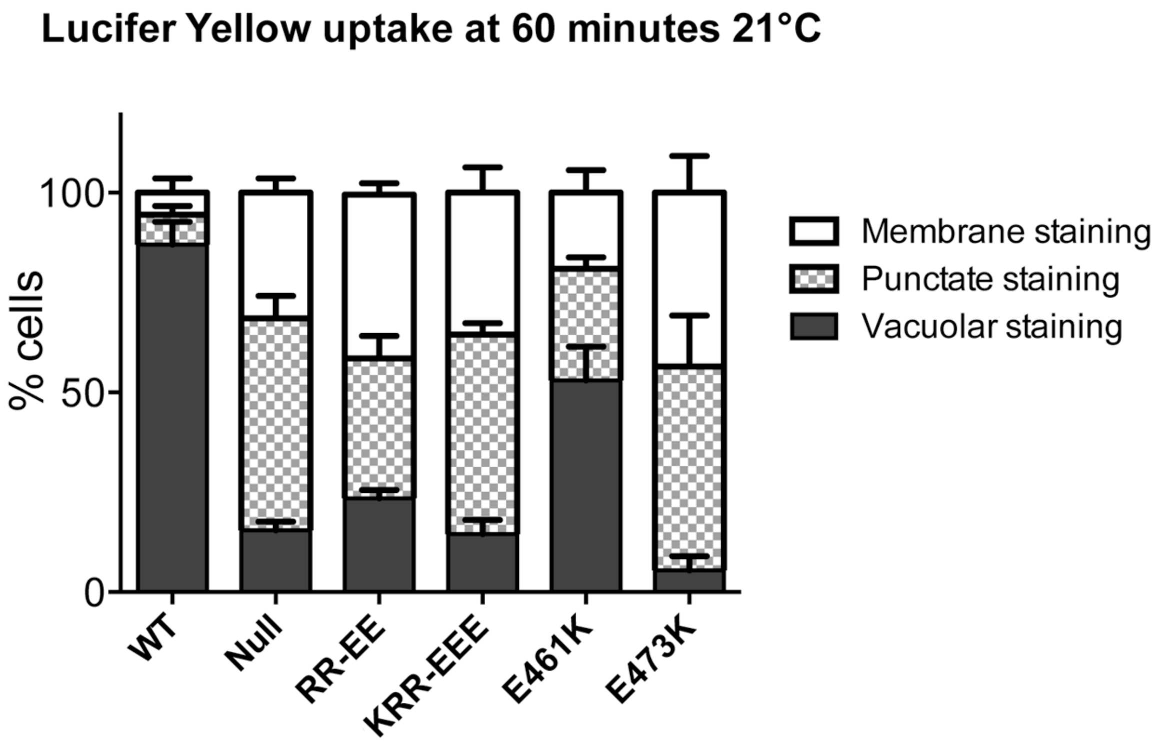

Lucifer yellow uptake was monitored for 90 minutes. Shown is the breakdown of predominant cell staining at the 60 minute time point for cells expressing *vps1* mutants as well as the wild type and deletion controls. Localization was classified as being predominantly vacuolar, endosomal or at the plasma membrane.

Figure S3 Bimolecular fluorescence complementation assay for Vps1 and Rvs167. Further support for Vps1 colocalization to endocytic sites in addition to that shown in Figure 3A.

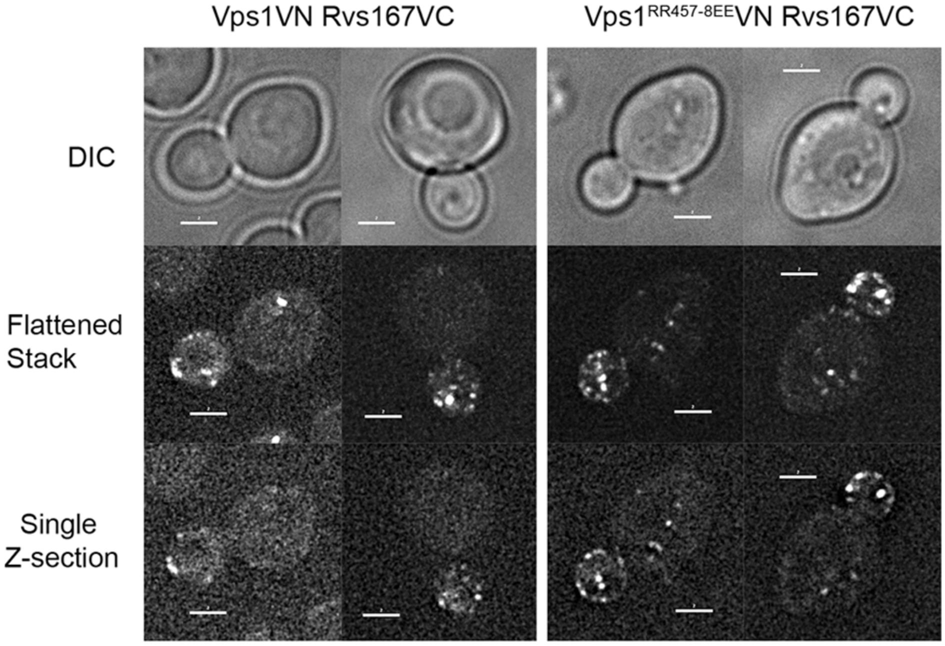

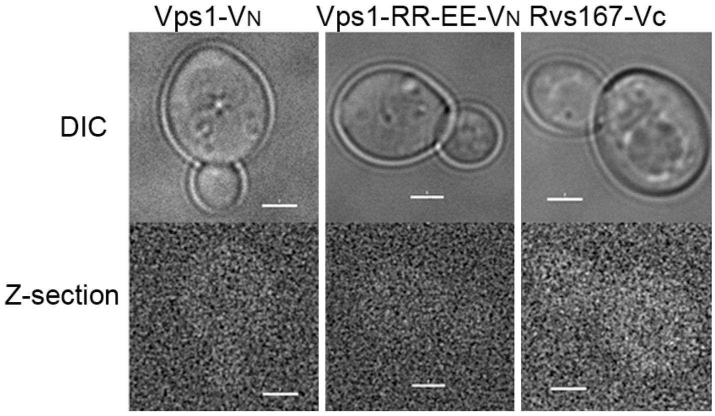

Vps1 and integrated vps1 mutants were tagged in the genome with sequence for N-terminus of Venus. The C-terminal region of Venus was integrated at the C-terminus of the amphiphysin gene Rvs167. Fluorescence signal indicates an interaction between the two split Venus proteins. Upper panels show two representative cells for cells expressing wild type Vps1 and the vps1 RR-EE mutant. Lower panels: control images of cells for the bimolecular fluorescence complementation assay expressing either Rvs167-V_N- or Vps1-V_C fusions demonstrating that there was no fluorescence signal when single fusions were present in cells.

Figure S4. Localization and Organization of Pil1-mRFP in wild type and vps1 RR-EE cells. Relates to possible identity of long invaginations shown in Figure 4.

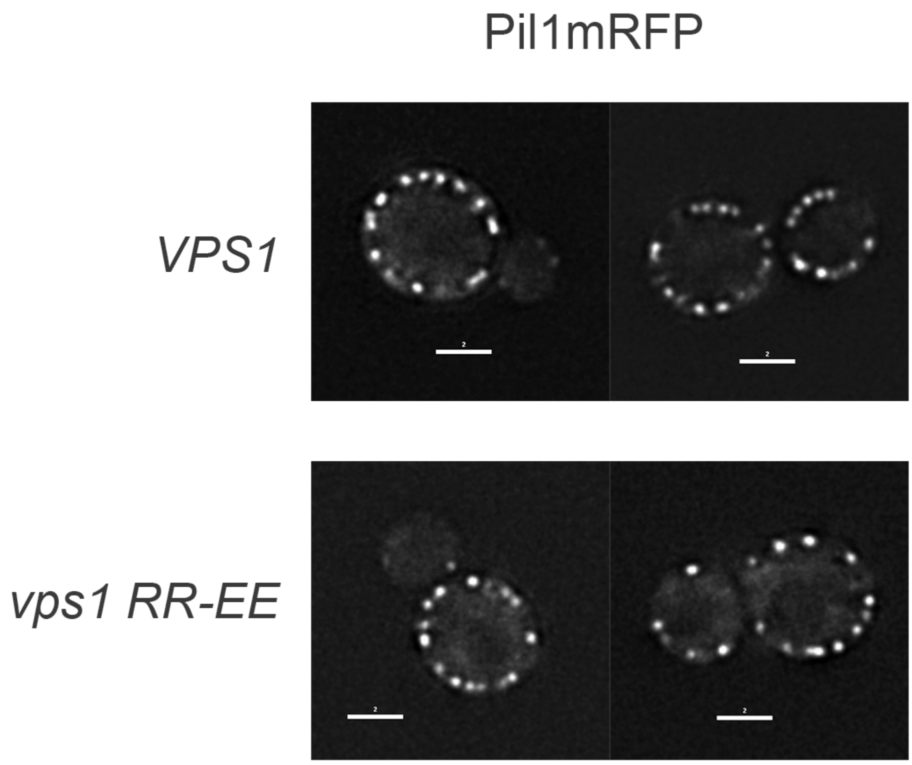

Strains KAY389 (*VPS1*) and KAY1793 (*vps1 RR-EE*) were transformed to integrate PCR generated mRFP tag sequence on the eisosomal component Pil1. Cells were imaged using Delatavision microscope and with 0.5 seconds exposure. Z-sections were taken and a single section is shown. No changes in Pil1 spot number, organization or localization were observed

Figure S5. Circular dichroism analysis for purified Vps1 and Vps1 RR-EE
Control data to determine any structural changes to proteins used to generate data in Figure 5.

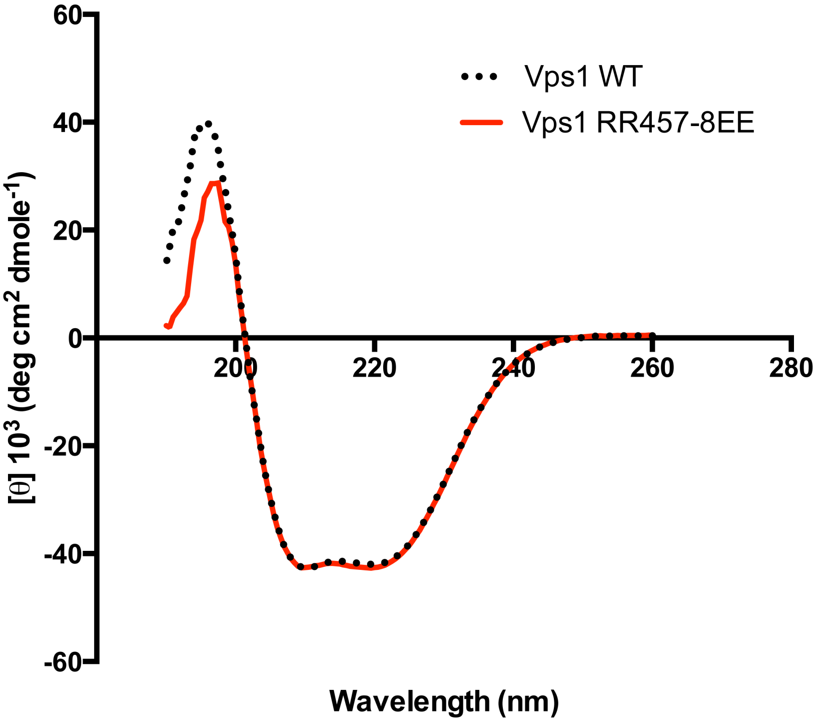

Far UV CD spectra were recorded on a Jasco J-810 spectropolarimeter at room temperature. Protein concentration was 2.5 μM. Black dashed line represents WT Vps1 with the red continuous line representing the RR457-8EE mutant. The spectra clearly show that the introduced mutations in the 457-8 position does not affect the secondary structure of the protein. Spectra of WT and the RR457-8EE mutant were taken with two separate purified proteins repeats and both have the same spectral profiles. The slight variation in spectra below 200 nm is considered to be due to different efficiencies in buffer exchange and does not reflect changes in folding.

Supplemental Tables

Table S1. Yeast strains used in this study

Strain number	Genotype	Notes
KAY302	*MATα ura3-52, leu2-3,112, his3Δ200, trp1-1*	KA lab
KAY389	*Mat a, ura3-52, leu2-3,112, his3Δ200, trp1-1, lys2-801*	KA lab
KAY1095	*MATα his3Δ1, leu2Δ0, lys2Δ, ura3Δ0 Δvps1::KanMx*	E.Hettema (Univ of Sheffield)
KAY1096	*MATα his3Δ1, leu2Δ0, lys2Δ, ura3Δ0 Δdnm1::KanMx Δvps1::HIS5*	E.Hettema (Univ of Sheffield)
KAY1459	*MATa SLA2-GFP::HIS3, his3Δ1, leu2Δ0, met15Δ0, ura3Δ0, vps1::LEU2*	[1]
KAY1337	*MATa Rvs167-GFP::HIS3, his3Δ1, leu2Δ0, met15Δ0, ura3Δ0 Δvps1::LEU2*	[1]
KAY1462	*MATα his3Δ1, leu2Δ0, lys2Δ, ura3Δ0 Δvps1::KanMx, GFP-Snc1-SUC2 URA3*	[1]
KAY1664	*MATa/α vps1::LEU2/vps1::KanMx, ABP1/Abp1-mCherry::HIS3, SLA1-GFP::HIS3/SLA1, his3Δ1/ his3Δ1, leu2Δ0/ leu2Δ0, met15Δ/MET15, LYS2/lys2Δ, ura3Δ/ura3Δ*	This study
KAY1756	*MAT a, his3-Δ200, leu2-3/112, ura3-52, trp1-1, lys2-801, vps1Δ::URA*	This study
KAY1793	*MAT a, his3-Δ200, leu2-3/112, ura3-52, trp1-1, lys2-801, vps1E461K*	This study
KAY1794	*MAT a, his3-Δ200, leu2-3/112, ura3-52, trp1-1, lys2-801, vps1RR457-8EE*	This study
KAY1806	*MAT a, his3-Δ200, leu2-3/112, ura3-52, trp1-1, lys2-801, vps1K453E, RR457-8EE*	This study
KAY1807	*MAT a, his3-Δ200, leu2-3/112, ura3-52, trp1-1, lys2-801, vps1E473K*	This study
KAY1466	*MATα his3Δ1, leu2Δ0, lys2Δ, ura3Δ0, Abp1-mCherry::HIS*	This study
KAY1621	*MATα, his3-Δ200, leu2-3/112, ura3-52, trp1-1, lys2-801, RVS167-VC::TRP1*	This study
KAY1832	*MAT a, his3-Δ200, leu2-3/112, ura3-52, trp1-1, lys2-801, VPS1-VN::HIS3*	This study

KAY1834	*MAT a, his3-Δ200, leu2-3/112, ura3-52, trp1-1, lys2-801, VPS1RR457-8EE-VN::HIS3*	This study
KAY1849	*Pil1mRFP in KAY 389 (Vps1 WT)*	This study
KAY1850	*Pil1mRFP in KAY 1793 (Vps1 RR-EE)*	This study

Table S2 – Plasmids used in this study

Plasmid number	Description	Origin
pKA 677	Vps1 under its own promoter for in vivo expression, *URA3*	[1]
pKA 943	pKA 677 with RR457-8EE, *URA3*	This study
pKA 944	pKA 677 with RR457-8EE+K453E, *URA3*	This study
pKA 945	pKA 677 with E461K, *URA3*	This study
pKA 946	pKA 677 with E473K, *URA3*	This study
pKA 850	His tagged Vps1 WT	[2]
pKA 969	pKA850 with *vps1 RR457-8EE*	This study
pKA 1025	pKA850 with *vps1 E461K*	This study
pKA 544	*URA* marked empty plasmid	E.Hettema (Univ of Sheffield)
pKA 1095	pKA850 with *vps1 E473K*	This study
pKA 1096	pKA850 with *vps1 RR457,458EE, K453E*	This study
pKA910	GFP-PTS_1 under *TPI1* promoter, *LEU2*	E.Hettema (Univ of Sheffield)
pKA1070	pVPS1-VPS1-GFP EcoR1-Pst1 from pKA836	[2]
pKA1101	pKA1070 with vps1 RR457,458EE	This study

Supplemental Experimental Procedures

Yeast strains, plasmids and cell growth. Yeast strains and plasmids used in this study are listed in Supplemental Tables, S1 and S2 respectively.

Integration of point mutation into the genome. The *VPS1* ORF, carrying a point mutation, was amplified by PCR together with the 50 bp flanking regions homologous to the sequences 5' from ATG and 3' from STOP codon. The amplified cassette was then transformed into *vps1Δ::URA3* strain. After growing overnight on YPD medium, the colonies were counter-selected on the minimum medium, containing 0.005% uracil and 0.1% 5-Fluoroorotic Acid (5-FOA; Melford laboratories). Allele exchange, in growing $Ura3^-$ 5-FOA resistant colonies, was confirmed by PCR and sequencing.

Preparation of Lipids and binding assays

25 mg/ml solution of Folch lipids (Avanti) in chloroform were dried with nitrogen then rehydrated in liposome buffer (20 mM HEPES pH7.2, 100 mM KCl, 2 mM $MgCl_2$, 1 mM DTT) to 20 mg/ml. Left for 1 hour at 60°C, freeze thawed in liquid nitrogen four times before snap freezing and storing at -20°C. In each co-sedimentation assay lipids were used at a concentration of 1 mg/ml. Following incubation with Vps1 wild-type or mutant protein, liposomes were spun at 90,000 rpm in a Beckman table top TLA-100 ultracentrifuge) for 15 minutes.

Circular Dichroism Analysis. All spectra were recorded on a Jasco J-810 spectropolarimeter. Protein was purified as described and buffer exchanged into 20 mM phosphate (pH 7.4) immediately before use. Buffer only measurements showed that the chosen buffer did not mask protein signal and was subtracted for baseline correction. Spectra were recorded as an average of three scans. Scan speed was 50 nm/min with a 1 second response time. Data was recorded using a 1 mm path length quartz cuvette.

Supplemental References

S1. Smaczynska-de Rooij, II, Allwood, E.G., Aghamohammadzadeh, S., Hettema, E.H., Goldberg, M.W., and Ayscough, K.R. (2010). A role for the dynamin-like protein Vps1 during endocytosis in yeast. Journal of Cell Science *123*, 3496-3506.

S2. Smaczynska-de Rooij, II, Allwood, E.G., Mishra, R., Booth, W.I., Aghamohammadzadeh, S., Goldberg, M.W., and Ayscough, K.R. (2012). Yeast Dynamin Vps1 and Amphiphysin Rvs167 Function Together During Endocytosis. Traffic *13*, 317-328.
